# Pre-clinical Evaluation of a Cyanine-Based SPECT Probe for Multimodal Tumor Necrosis Imaging

**DOI:** 10.1007/s11307-016-0972-7

**Published:** 2016-06-08

**Authors:** Marieke A. Stammes, Vicky T. Knol-Blankevoort, Luis J. Cruz, Hans R. I. J. Feitsma, Laura Mezzanotte, Robert A. Cordfunke, Riccardo Sinisi, Elena A. Dubikovskaya, Azusa Maeda, Ralph S. DaCosta, Katja Bierau, Alan Chan, Eric L. Kaijzel, Thomas J. A. Snoeks, Ermond R. van Beek, Clemens W. G. M. Löwik

**Affiliations:** 1 Department of Radiology, Leiden University Medical Center, Leiden, The Netherlands; 2 Percuros BV, Leiden, The Netherlands; 3 Department of Nuclear Medicine, Leiden University Medical Center, Leiden, The Netherlands; 4 Department of Radiology, Erasmus Medical Center, Building-room: Na606, Wytemaweg 80, 3015 CN Rotterdam, The Netherlands; 5 Department of Clinical Pharmacy and Toxicology, Leiden University Medical Center, Leiden, The Netherlands; 6 Institute of Chemical Sciences and Engineering (ISIC), École Polytechnique Fédérale de Lausanne (EPFL), Lausanne, Switzerland; 7 Division of Biophysics and Bioimaging, Princess Margaret Cancer Center, University Health Network, Toronto, Ontario Canada; 8 Department of Surgery, Leiden University Medical Center, Leiden, The Netherlands

**Keywords:** Radiolabeling, Multimodal imaging, Cyanine, Necrosis avid contrast agent, Cancer

## Abstract

**Purpose:**

Recently we showed that a number of carboxylated near-infrared fluorescent (NIRF) cyanine dyes possess strong necrosis avid properties *in vitro* as well as in different mouse models of spontaneous and therapy-induced tumor necrosis, indicating their potential use for cancer diagnostic- and prognostic purposes. In the previous study, the detection of the cyanines was achieved by whole body optical imaging, a technique that, due to the limited penetration of near-infrared light, is not suitable for investigations deeper than 1 cm within the human body. Therefore, in order to facilitate clinical translation, the purpose of the present study was to generate a necrosis avid cyanine-based NIRF probe that could also be used for single photon emission computed tomography (SPECT). For this, the necrosis avid NIRF cyanine HQ4 was radiolabeled with ^111^indium, via the chelate diethylene triamine pentaacetic acid (DTPA).

**Procedures:**

The necrosis avid properties of the radiotracer [^111^In]DTPA-HQ4 were examined *in vitro* and *in vivo* in different breast tumor models in mice using SPECT and optical imaging. Moreover, biodistribution studies were performed to examine the pharmacokinetics of the probe *in vivo*.

**Results:**

Using optical imaging and radioactivity measurements, *in vitro*, we showed selective accumulation of [^111^In]DTPA-HQ4 in dead cells. Using SPECT and in biodistribution studies, the necrosis avidity of the radiotracer was confirmed in a 4T1 mouse breast cancer model of spontaneous tumor necrosis and in a MCF-7 human breast cancer model of chemotherapy-induced tumor necrosis.

**Conclusions:**

The radiotracer [^111^In]DTPA-HQ4 possessed strong and selective necrosis avidity *in vitro* and in various mouse models of tumor necrosis *in vivo*, indicating its potential to be clinically applied for diagnostic purposes and to monitor anti-cancer treatment efficacy.

**Electronic supplementary material:**

The online version of this article (doi:10.1007/s11307-016-0972-7) contains supplementary material, which is available to authorized users.

## Introduction

Necrosis is a form of cell death characterized by severe cell swelling, denaturation and coagulation of cytoplasmic proteins, and disruption of the cell membrane, causing the release of its intracellular content. Necrotic cell death is irreversible and is induced by external factors or disease, such as radiation, trauma, and loss of blood supply, and is also involved in cancer development and treatment [1, 2]. In the center of most solid tumors, an area of ischemia and subsequent necrosis develops, as vascularization cannot keep up with the rapidly growing tumor mass. The size and growth rate of this necrotic area is positively correlated with the aggressiveness of cancer and can, therefore, be used as a diagnostic biomarker of cancer staging [3–8]. Moreover, anti-cancer treatments like chemotherapy are utilized to induce cell death, increasing the total amount of tumor necrosis [9–11]. Thus, agents that specifically bind to necrotic tumor tissue can contribute to a more accurate disease diagnosis and can be exploited to predict early treatment outcome of anti-cancer treatments [12]. To this end, back in 1988, Epstein and colleagues [13] developed so-called tumor necrosis targeting (TNT) antibodies that are directed towards nuclear proteins and labeled with radioactive iodine for imaging and anti-cancer treatment purposes. Likewise, the photosensitizing agent hypericin has also been shown to possess necrosis avidity and is currently under investigation for cancer imaging and treatment purposes [14–17]. However, there are several drawbacks for the existing agents. Antibodies are relatively large in size, have long circulation time, could induce an immune response, and expensive to develop in good manufacturing practices (GMP) quality, and the photosensitizer hypericin is phototoxic, poorly soluble, and tend to aggregate rapidly [16, 18, 19]. All these issues hamper the clinical translation of these compounds [14, 20–22].

Recently, we reported on two near-infrared fluorescent (NIRF) carboxylated cyanine dyes, HQ5 and IRDye 800CW (800CW), that also possess strong necrosis avid properties [23]. Cyanines are, among other dyes, widely used as fluorescent tags for protein labeling to enable whole body optical imaging in small animals [24–28]. Although the mechanism of necrosis avidity is not fully understood, it is independent of the enhanced permeability and retention (EPR) effect [23, 29]. On a cellular level, increased retention of the dye may involve augmented accessibility to cells that have lost membrane integrity along with an increased affinity to denatured cytoplasmatic proteins [30–32]. Using whole body optical imaging in mice, we showed that these cyanines can be employed to image areas of spontaneous necrosis in solid tumors and to determine the efficacy of chemotherapy by monitoring therapy-induced tumor necrosis [23].

Optical imaging is successfully applied in whole body imaging for small animals and in imaging of superficial tissues in humans. Optical imaging is based on the detection of light emitted from agents and/or tags coupled to biomolecules in living systems. In order to obtain better tissue penetration, optical imaging often makes use of agents and tags that emit light in the near-infrared range. However, even with these compounds, the maximal penetration depth is limited to a 1-2 centimeters [33, 34]. Although this relatively small measuring range is sufficient for the detection of light in small animals, it is not sufficient for the detection of light in deep tissues such as tumors situated deep within the human body. Alternatively, nuclear imaging modalities such as single photon emission computed tomography (SPECT) and positron emission tomography (PET) are used clinically to image deep tissues. To enable SPECT and/or PET for clinical translation, our previously described necrosis avid NIRF cyanines must be radiolabeled. Generally, this is achieved by conjugating a targeting moiety to a chelate that is subsequently labeled with a metal radionuclide [35–38]. In the present study we employed this principle to the necrosis avid cyanine HQ4, a close structural analogue of the recently studied necrosis avid cyanine HQ5 [23]. As HQ5 has two moieties which can be functionalized by the chelate diethylene triamine pentaacetic acid (DTPA), we used HQ4, a mono-derived analogue, to avoid scrambling of the DTPA molecules or steric hindrance [39]. HQ4 was conjugated to the chelate (DTPA) and subsequently labeling with ^111^indium-chloride (^111^In-Cl_3_) was performed. In this study, we investigated the necrosis avidity of [^111^In]DTPA-HQ4 *in vitro* and *in vivo * in mouse tumor models of spontaneous and chemotherapy-induced tumor necrosis using optical imaging and SPECT.

## Material and Methods

### Compounds

The cyanine dyes, HQ4 carboxylate and HQ4-NHS ester, were obtained from Ilumicare BV (Rotterdam, The Netherlands).

### Synthesis of HQ4-DTPA

Synthesis of DTPA-polyethylene glycol (PEG)-NH_2_, DTPA containing PEG amine link (4,7,10-trioxa-1,13-tridecanediamine), indicated as PEG-NH_2_ in the formula (DTPA-PEG-NH_2_) was synthesized on chloride-trityl chloride (Cl-TrtCl) resin. Thus, fluorenylmethyloxycarbonyl (Fmoc)-PEG amine was incorporated on Cl-TrtCl resin (CTC resin) by reacting 3 eq. Fmoc-PEG amine in presence of 6 eq. *N*,*N*-diisopropylethylamine (DIEA) in dichloromethane (DCM) overnight at room temperature (RT). Final loading was measured by Fmoc quantification and the value obtained was around 0.8 mmol/g. Fmoc group removal was carried out with piperidine–dimethylformamide (DMF) (1:5) (1 × 1 min, 2 × 10 min). Next, the DTPA-(terta-tBu ester)-COOH (2 eq.) was coupled using *N*,*N*′-dipropan-2-ylmethanediimine (DIPCDI) (2 eq.) and hydroxybenzotriazole (HOBt) (2 eq.) in DMF overnight. After coupling overnight, the ninhydrin test was negative. Later on, DTPA-(terta-tBu ester)-CO-NH-PEG-CTC-resin cleavage and deprotection was performed in two steps. DTPA-(terta-tBu ester)-CO-NH-PEG-CTC-resin was treated with 1 % trifluoroacetic acid (TFA) in DCM 10 times for 1 min each time. Excess DCM was removed using vacuum and side chain protecting groups were removed using 95 % TFA, 2.5 % triisopropylsilane (TIS), and 2.5 % water. DTPA-CO-NH-PEG-NH_2_ was precipitated with cold methyl-tert-butylether (MTBE) after TFA removal under a N_2_ stream. The DTPA-CO-NH-PEG-NH_2_ was dissolved in water and lyophilized to obtain the final product. The desired DTPA-CO-NH-PEG-NH_2_ was 85.0 % in yield with a purity of 90.6 % as analyzed by high-performance liquid chromatography (HPLC) (retention time 2.28 min). HPLC–mass spectrometry (MS), *m*/*z* calc.: 523.25 for C_20_H_37_N_5_O_11_, Found: 524.5.28 [M+1]+ and matrix-assisted laser desorption/ionization time-of-flight (MALDI-TOF) analysis found 524.2 [M+1]+546.3 [M+Na].

Synthesis of DTPA-CO-NH-PEG-NH_2_–HQ4: HQ4-NHS (8.3 × 10^−4^ mmol, 1 mg) dissolved in 50 μl of dimethyl sulfoxide (DMSO) was added to DTPA-CO-NH-PEG-NH_2_ (2.8 × 10^-3^ mmol, 2 mg) dissolved in 200 μl of DMSO containing 5 μl DIEA and stirred overnight at room temperature. Later on, the complex DTPA-CO-NH-PEG-HQ4 was purified by reversed phase (RP)-HPLC. The desired DTPA-PEG-HQ4 was 50.5 % in yield with a purity of 98 % as analyzed by HPLC (tR 5.34). HPLC-MS, *m*/*z* calc.: 1331.59 for C_66_H_90_N_8_O_17_S_2_, Found: 1332.0[M+1]+ and MALDI-TOF Found 1331.9 [M+1]+ 1353.9 [M+Na].

### Cells and Culture Conditions

4T1-luc2 mouse mammary cancer cells (PerkinElmer, Waltham, MA, USA) and MCF-7 human mammary cancer cells were all cultured in RPMI-1640 medium (Life Technologies Inc., Carlsbad, CA, USA) supplemented with 10 % fetal calf serum (FCS; Lonza, Basel Switzerland), 100 units/ml penicillin, and 50 μg/ml streptomycin (Life Technologies Inc.). All cell lines were cultured in a humidified incubator at 37 °C and 5 % CO_2_, monthly checked for *Mycoplasma* infection by polymerase chain reaction (PCR) and checked routinely for morphologic changes.

### Dry Ice Dead Cell Assay

*In vitro*, cell death was studied using a cryo-induced cell death assay as previously described [40]. In short, 4T1-luc2 cells were seeded onto 24-well tissue culture plates (Sigma-Aldrich) and grown until confluent. After discarding the medium, a bar of dry ice 3–5 mm in diameter was applied to the underside of the culture well for 20 s. Subsequently, the cells were incubated in the dark for 15 min at RT with HQ4 at a concentration of 100 nM. After incubation, the samples were gently washed with phosphate-buffered saline (PBS) and subsequently scanned for fluorescence using the Odyssey Infrared Imager 9120 (LI-COR) and for radioactivity via phosphor imaging on the Typhoon 9410 imager (GE Healthcare).

### *In Vitro* Viability Assay

4T1-luc2 cells were plated in a 96-well plate (Costar) in 100 μl medium at a density of 10,000 cells per well and left over night to adhere. The next day, the medium was replaced with medium containing the experimental compounds: HQ4, HQ4-DTPA, HQ5, and Gambogic acid (GA), three wells per condition. After 24 h, cell viability was measured using a nonradioactive colorimetric MTS viability assay (Promega Benelux) according to the manufacturer’s protocol. Optical absorption was measured at 490 nm with a Versamax absorbance microplate reader (Molecular Devices).

### Animals

Female athymic mice (BALB/c nu/nu, 6 weeks old) were purchased from Charles River Laboratories (L′Arbresle Cedex, France). Animals were housed per four to five animals in individually ventilated cages at 22 °C and 50 % humidity with free access to food and water and maintained under standard 12 h light/12 h dark cycles. All surgical and analytical procedures were performed under isoflurane gas anesthesia (3 % induction, 1.5–2 % maintenance) in 70 % pressurized air and 30 % O_2_. Animals were sacrificed by cervical dislocation at the end of the experimental period.

All animal experiments were assessed for animal health, ethics, and research and approved by the Animal Welfare Committee of Leiden University Medical Center, the Netherlands. All mice received humane care and were kept in compliance with the Code of Practice Use of Laboratory Animals in Cancer Research (Inspectie W&V, July 1999).

### Subcutaneous Tumor Model

Approximately 1 × 10^5^ 4T1-luc2 cells, suspended in 15 μl PBS, were implanted bilateral and subcutaneously onto the upper back of nude mice. Tumors were grown until they reached a size of 6–7 mm in diameter which developed roughly after 1.5–2 weeks of tumor implantation.

Similarly, 2 × 10^6^ MCF-7 cells, suspended in 15 μl PBS in a 1:1 mixture with 15 μl of Matrigel (BD biosciences, San Jose, CA, USA), were implanted bilateral and subcutaneously onto the upper back. Tumors were grown until they reached 6–7 mm diameter, which developed after approximately 2–3 weeks.

Whole body FLI measurements were performed using the Pearl Impulse Small Animal Imaging System (LI-COR) and/or IVIS Spectrum *in vivo* imaging system (PerkinElmer) several time points after injection. On the IVIS, an excitation and emission wavelength of 675 and 720 nm was used for HQ4.

### Radiolabeling of HQ4-DTPA and SPECT

To label HQ4-DTPA with ^111^In-Cl_3_, it was dissolved in 0.1 M Hepes (10 μg/100 μl) [41] and ^111^In-Cl_3_ (35 MBq; Covidien-Mallinckrodt, Petten, The Netherlands) was added. After 30 min of incubation on the shaker, labeling was validated with HPLC (Jasco Inc., Easton, MD, USA) or thin layer chromatography (TLC). In all cases, labeling efficacy was >90 %.

To study the specificity of the radiolabeled HQ4-DTPA versus radiolabeled DTPA *in vivo*, 10 μg [^111^In]DTPA-HQ4 (=10 nmol per mouse) or 10 μg [^111^In]DTPA was injected i.v. into mice bearing 4T1-luc2 tumors (*n* = 3 and 4, respectively). The total injected dose (ID) in each mouse was determined in a dose-calibrator (VDC101, Veenstra Instruments, Joure, the Netherlands).

SPECT scans were conducted at several time points post injection on a three-headed U-SPECT-II gamma camera (MILabs, Utrecht, The Netherlands) under isoflurane anesthesia for 40 min. Radioactivity counts from total body scans were acquired using a 0.6-mm mouse pinhole collimator with energy settings at 171 and 245 keV with a window of 20 % and background energy settings of respectively 4.5 and 3.5 % around the tails of the energy window [42]. Subsequently, the image was reconstructed using 20 POSEM iterations with four subsets, 3D gauss 1 mm (FWHM) filtering, a 0.2-mm voxel size, and with decay and scatter corrections integrated into the reconstruction [43]. Images were generated and analyzed using PMOD software. *In vivo* SPECT was followed by *in vivo* fluorescence imaging on the Pearl Impulse Small Animal Imaging System (LI-COR).

After the last imaging time point, mice were sacrificed and several tissues were excised, weighed, and counted for radioactivity (Wizard2 2470 automatic gamma scintillation counter, Perkin Elmer, USA) to determine the percentage of the injected dose per gram (%ID/g). The %ID/g was calculated as follows: (((MBq measured in tissue/injected dose) × 100 %) / weight of tissue).

### Chemotherapy of MCF-7 Tumors

In two groups of mice (*n* = 5), 2 weeks after tumor implantation the mice received either an intraperitoneal (i.p.) injection of cyclophosphamide (265 mg/kg; Baxter BV, Utrecht, The Netherlands) [44] or remained untreated. After 72 h, all animals received an i.v. injection of radiolabeled HQ4-DTPA. SPECT scans were conducted 24 h later, followed by whole body FLI. After the last imaging time point, mice were sacrificed, several tissues were excised, weighted, and counted for radioactivity.

### *Ex Vivo* Tumor Imaging

*Ex vivo* tumor imaging was performed to visualize the distribution of the probe in the tumor. Images were obtained for FLI (Odyssey Infrared Imager 9120 (LI-COR)) and for radioactivity via phosphor imaging (Typhoon 9410 imager (GE Healthcare) and ImageQuant TL software). For phosphor imaging, the tumors were manually sliced in sagittal sections and placed o/n on a phosphor screen.

### Histological Procedures

The tumors collected for histological analysis were fixed in 4 % formaldehyde and embedded in paraffin. Five micrometer sections were prepared and FLI was performed using the Odyssey Infrared Imager (LI-COR). Afterwards, the sections were subjected to TdT-mediated dUTP Nick-End Labeling (TUNEL) staining (Promega, Madison, WI, USA) to validate accumulation of the NIRF probes in necrotic cells.

### Statistical Analysis

All statistical analysis was performed using Prism software (GraphPad). For repeated measures, a Student’s *t* test was used in all cases. *P* < 0.05 was considered significant, and all error bars represent mean $\pm$ SEM.

## Results

We selected the necrosis avid cyanine HQ4, instead of HQ5, to perform radiolabeling and further *in vitro* and *in vivo* investigations to demonstrate its potential clinical translation. The choice for HQ4 was based on the number of functionalized moieties present in the cyanine molecules, which is one in the case of HQ4 and two in the case of HQ5 (see Online Resource 1, suppl. Fig. 1a). Furthermore, as shown in online Resource 1, suppl. Fig. 1b, HQ4 showed slightly higher necrosis avid properties in our dry ice dead cell assay than HQ5. The fluorescent signal intensity, obtained from the area of cell death caused by freezing, was over the whole dose range (1–100 nM) 2.8 (+/−1.0)-fold higher for HQ4 as compared to HQ5.

**Fig. 1:**
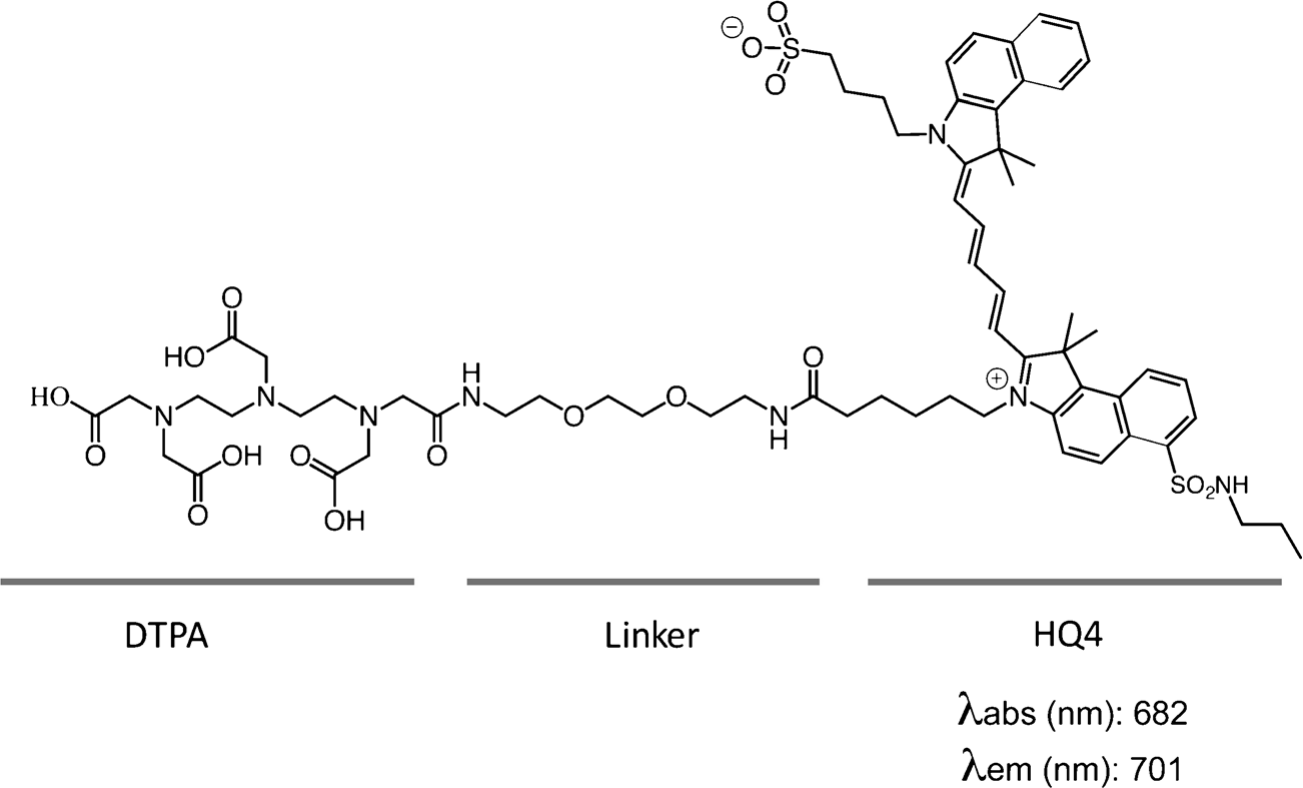
Chemical and structural characteristics of HQ4-DTPA. Chemical structure of HQ4-DTPA. *λ*_abs_ = absorbance wavelength; *λ*_em_ = emission wavelength.

A HQ4-DTPA hybrid complex was synthesized by covalent coupling via conjugation with a polyethylene glycol (PEG) linker (Fig. 1). Reversed-phase chromatography showed a clear peak indicating the high grade of purity (98 %) of this conjugate. Mass spectrometric analyses of HQ4-DTPA further showed the expected molecular weight (calc.: 1331.59 for C_66_H_90_N_8_O_17_S_2_ and MALDI-TOF found 1332.4 [M+1]+ 1354.6 [M+Na]), indicating the high grade of purity of this conjugate (see Online Resource 1, suppl. Fig. 2). Lastly, the HQ4-DTPA conjugate was labeled with ^111^In-Cl_3_ with a labeling efficiency of >90 % (data not shown).

### *In Vitro* Viability Assay

Using the MTS cell toxicity assay, the acute *in vitro* toxicity of the cyanine dyes HQ4, HQ4-DTPA, and HQ5 were examined in 24 h cultures of 4T1 breast cancer cell and compared with the cytotoxic compound GA. In the dose range of 0–20 μM, none of the cyanine compounds affected cell viability. GA, however, dose dependently induced cell death with an IC50 of around 6 μM (see Online Resource 1, suppl. Fig. 3).

### *In Vitro* Necrosis Targeting Properties of [^111^In]DTPA-HQ4

**Fig. 2:**
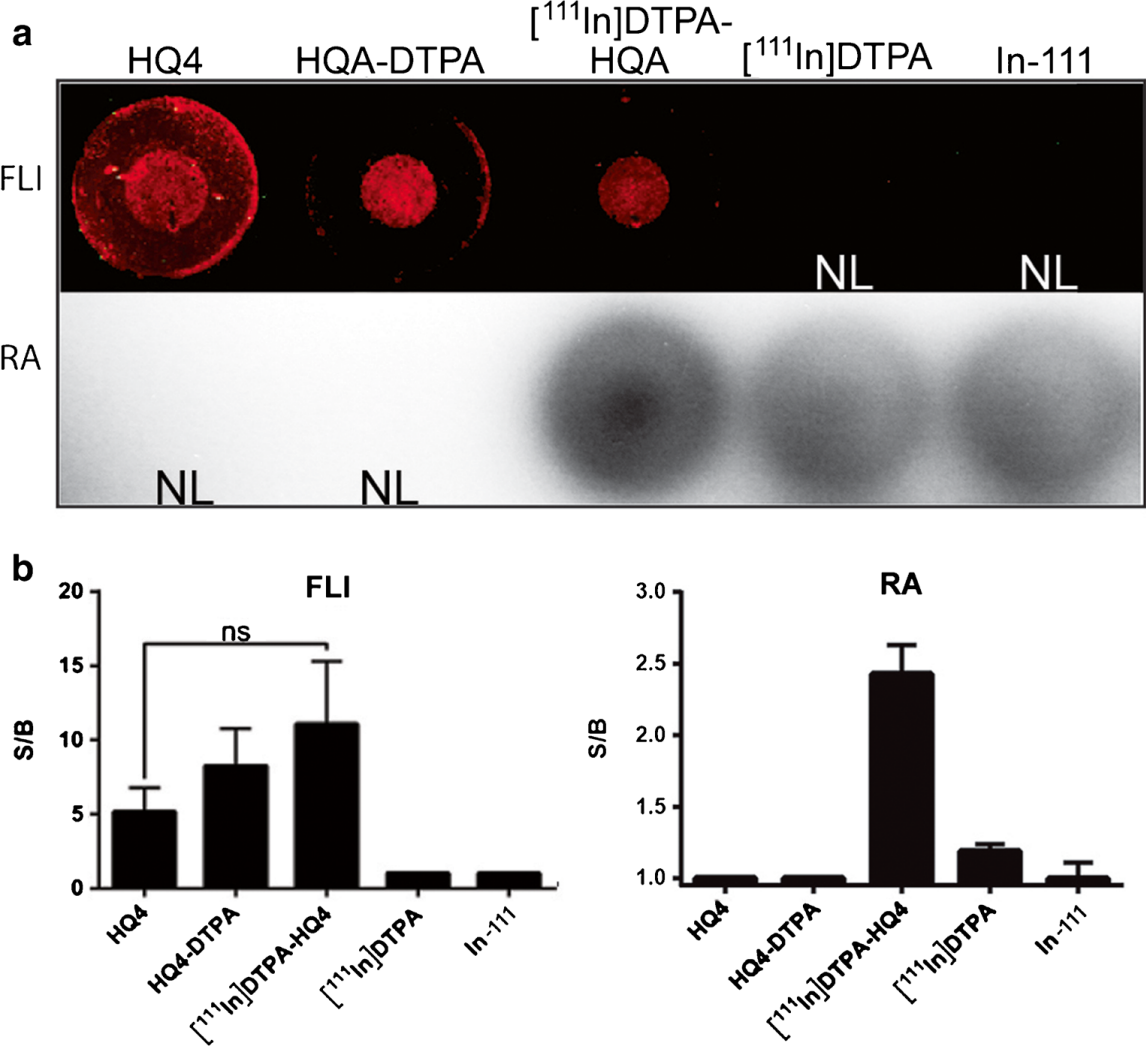
*In vitro* necrosis avid properties of [^111^In]DTPA-HQ4 by fluorescence and radioactivity measurements. **a** Necrotic cell death in confluent monolayers of 4T1 mouse breast cancer cells was induced by applying dry ice to the underside of the culture well. Cells were subsequently incubated for 15 min with one of the following: HQ4, HQ4-DTPA, [^111^In]DTPA-HQ4, [^111^In]DTPA, or ^111^In-Cl_3_ (100 nM, 10 μg). After subsequent washing with PBS, cells were imaged either for fluorescence (*FLI*, *upper panel*) or for radioactivity (*RA*, *lower panel*). *NL* = not labeled. **b** Calculated signal-to-background (S/B) ratio (FLI and RA) of the different compounds obtained from the dry ice assay. The S/B ratio was defined as the signal intensity obtained from the area of dead cells in the center of the well divided by the signal intensity obtained from an area of living cells of the same size in the periphery.

The dry ice dead cell assay was performed to examine the necrosis avid properties of the entire conjugate [^111^In]DTPA-HQ4 and the individual components, HQ4 and HQ4-DTPA, respectively, *in vitro*. HQ4, HQ4-DTPA, and [^111^In]DTPA-HQ4 all strongly accumulated in the area of dead cells, as shown by fluorescence imaging (Fig. 2a). In addition, radioactive signal was obtained in areas of dead cells only when incubated with [^111^In]DTPA-HQ4. In contrast, neither [^111^In]DTPA nor free ^111^In-Cl_3_ accumulated in the dead cells area, as indicated by RA measurements. Fig. 2b shows quantifications of the fluorescent and radioactive signal-to-background (S/B) (dead cells vs living cells area) ratios for the five different compounds tested confirming that [^111^In]DTPA-HQ4 retains fluorescence and radioactivity, as well as necrosis avidity with a S/B ratio of 11.1 and 2.4, respectively.

### Necrosis Targeting Properties of [^111^In]DTPA-HQ4 in a 4T1 Mouse Tumor Model of Spontaneous Necrosis

**Fig. 3:**
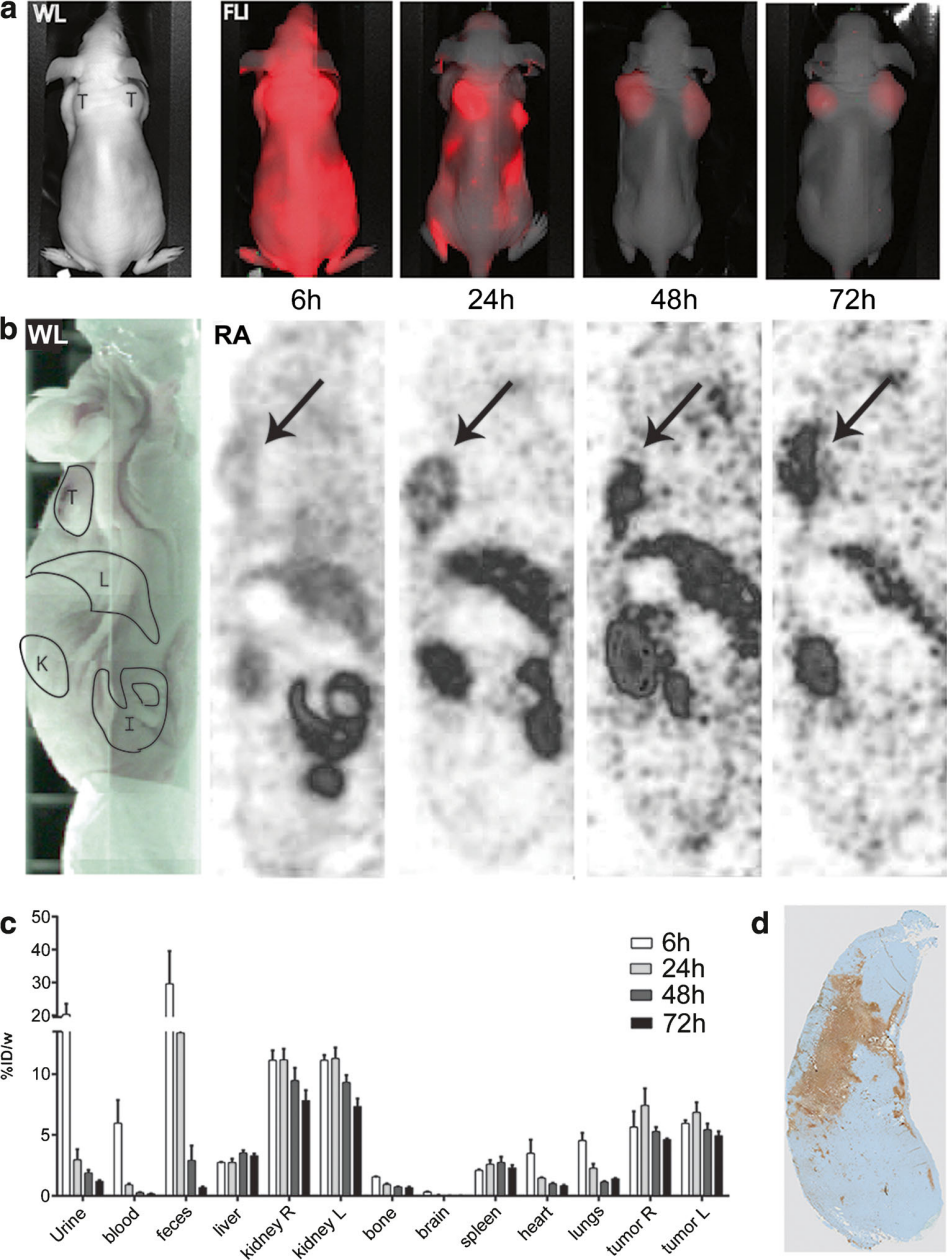
Optical imaging, SPECT, and biodistribution of [^111^In]DTPA-HQ4 in 4T1 breast tumor bearing mice. **a** Whole body FLI (coronal view) obtained 6–72 h after tail vein injection of [^111^In]DTPA-HQ4 (10 nmol, 30–35 MBq). The white light image (*WL*) indicates the position of the mouse in the Pearl imager, from a dorsal point of view (*T* = tumor). The same device settings were applied to all FLI, rendering comparison between the different images possible. **b** Whole body SPECT images (sagittal view) obtained 6–72 h after tail vein injection of [^111^In]DTPA-HQ4 (10 nmol, 30–35 MBq). The white light image (*WL*) indicates the position of the mouse from a sagittal point of view, in the SPECT (*RA*); *T* = tumor, *L* = liver, *K* = kidney, and *I* = intestine. *Arrows* indicate the tumor. **c** Biodistribution of [^111^In]DTPA-HQ4 in 4T1 tumor bearing mice. Six, 24, 48, and 72 h after probe injection, mice (*n* = 3 per time point) were sacrificed and the organs, body fluids, and tumors were dissected, weighed, and measured for radioactivity in a gamma counter. At each time point, the amount of radioactivity in the organs is expressed as percentage of the injected dose divided by body weight (%ID/w). **d** TUNEL-stained histological section of a representative 4T1 mouse breast tumor showing a large area of necrosis (*brown*).

The necrosis targeting properties of [^111^In]DTPA-HQ4 were evaluated in subcutaneous 4T1 breast tumor bearing mice, using SPECT and optical imaging. [^111^In]DTPA-HQ4 (10 nmol, 30–35 MBq) was injected intravenously (i.v.) into the tumor bearing mice, and *in vivo* SPECT and optical images were obtained 6, 24, 48, and 72 h, post injection (Fig. 3a, b). An increased retention of [^111^In]DTPA-HQ4 in the tumors and in the metabolizing organs was observed over time with both optical imaging and SPECT.

To further assess the biodistribution of [^111^In]DTPA-HQ4 in different organs, mice (*n* = 3) were sacrificed at each time point. Subsequently, radioactivity in various organs and body fluids was quantified (Fig. 3c). The measured radioactivity across different time points confirmed accumulation of radioactivity in the tumors (4.8 %ID/w), liver (3.3 %ID/w), and the kidneys (7.6 %ID/w) at 72 h post injection. Finally, histological examination of the 4T1 tumors confirmed the presence of a large necrotic core (Fig. 3d).

Similarly, the biodistribution of the radiolabeled chelate [^111^In]DTPA was examined in 4T1 tumor bearing mice 24 h after i.v. injection of [^111^In]DTPA (10 μg, 30–35 MBq). Mice (*n* = 4) were euthanized and the internal organs and body fluids were removed to quantify remaining radioactivity (see Online Resource 1, suppl. Fig. 4a). Greatest accumulation of radioactivity was observed in the kidneys (5.0 %ID/w) and only relatively low values of radioactivity could be measured in other organs, body fluids, and tumors (tumors 0.5 %ID/w, liver 0.6 %ID/w).

Supplementary Fig. 4b, Online Resource 1, shows the measured amount of radioactivity in the mouse corpus at different time points after injection of [^111^In]DTPA-HQ4 or [^111^In]DTPA, expressed as the percentage of the total injected doses (%ID). It was found that 24 and 48 h after injection of [^111^In]DTPA-HQ4, respectively, 38 and 35 % of total injected dose was retained in the body, whereas this was only 10 and 8 %, for [^111^In]DTPA.

### SPECT and Optical Imaging of [^111^In]DTPA-HQ4 in a MCF-7 Mouse Model of Chemotherapy-Induced Tumor Necrosis

**Fig. 4:**
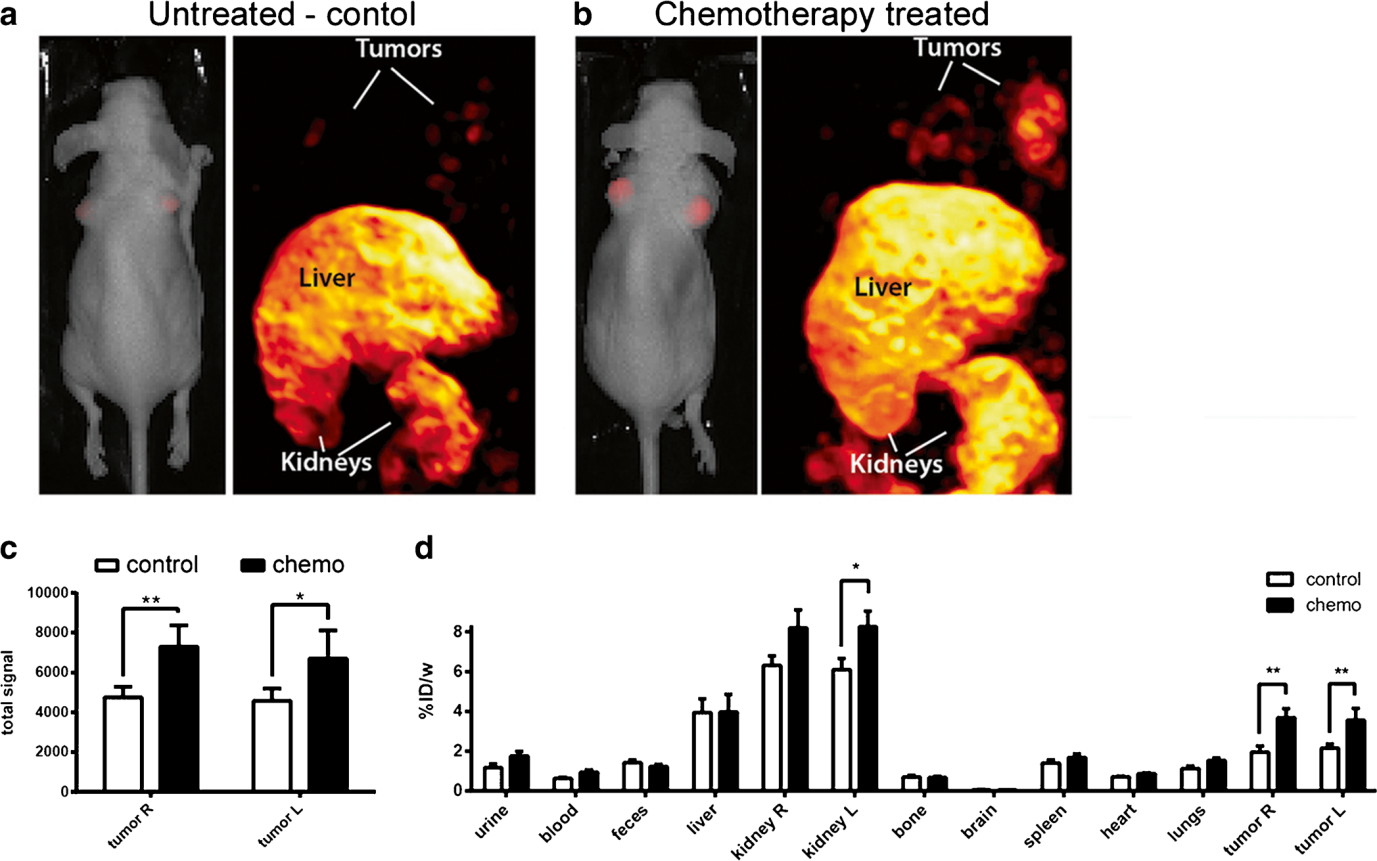
Optical imaging, SPECT, and biodistribution of [^111^In]DTPA-HQ4 in chemotherapy-treated MCF-7 tumor bearing mice. Representative whole body FLI (coronal) and SPECT (MIP) images of MCF-7 tumor bearing **a** control and **b** cyclophosphamide-treated mice. Mice were injected with [^111^In]DTPA-HQ4 (10 nmol, 30–35 MBq) 72 h after injection with chemotherapy. Whole body FLI and SPECT were acquired 24 h after probe injection. **c** Mean FL signal intensity obtained from the tumors of control and chemotherapy-treated mice (*n* = 5, two tumors per mouse), 24 h after injection of [^111^In]DTPA-HQ4. **d** Biodistribution of [^111^In]DTPA-HQ4 in MCF-7 tumor bearing mice. Mice were injected with [^111^In]DTPA-HQ4 72 h after chemo-treatment and were sacrificed 24 h later, and the organs, body fluids and tumors were dissected, weighed, and measured for radioactivity in a gamma counter. The amount of radioactivity in the organs is expressed as percentage of the injected dose divided by the weight (%ID/w).

Using SPECT and optical imaging, we examined whether [^111^In]DTPA-HQ4 could be employed to monitor chemotherapy-induced tumor necrosis. For this, MCF-7 tumor bearing mice were treated with a single i.p. injection of cyclophosphamide (265 mg/kg) [44], followed by an i.v. injection of [^111^In]DTPA-HQ4 (10 nmol, 30–35 MBq) 72 h later. Twenty-four hours after injection of [^111^In]DTPA-HQ4, whole body optical images and SPECT were obtained to assess its biodistribution. Both optical imaging and SPECT showed accumulation of [^111^In]DTPA-HQ4 in cyclophosphamide-treated tumors as compared to untreated tumors (Fig. 4a, b). Fig. 4c shows quantification of the fluorescent signals obtained from the tumors, 24 h after probe injection. A significant increase in tumor fluorescent signal intensity was observed in mice that were treated with chemotherapy as compared to untreated controls (ratio = 1.8:1.0, *p* = 0.0011).

Biodistribution study based on quantification of radioactivity in various organs further demonstrated higher amount of radioactivity in chemotherapy-treated tumors compared to untreated controls. The average %ID/w was 1.85 for the control tumors vs 4.02 for the chemo-treated tumors (Fig. 4d). In addition, a significantly higher amount (1.4-fold) of radioactivity was observed in left kidneys of the chemotherapy-treated mice as compared to controls.

**Fig. 5:**
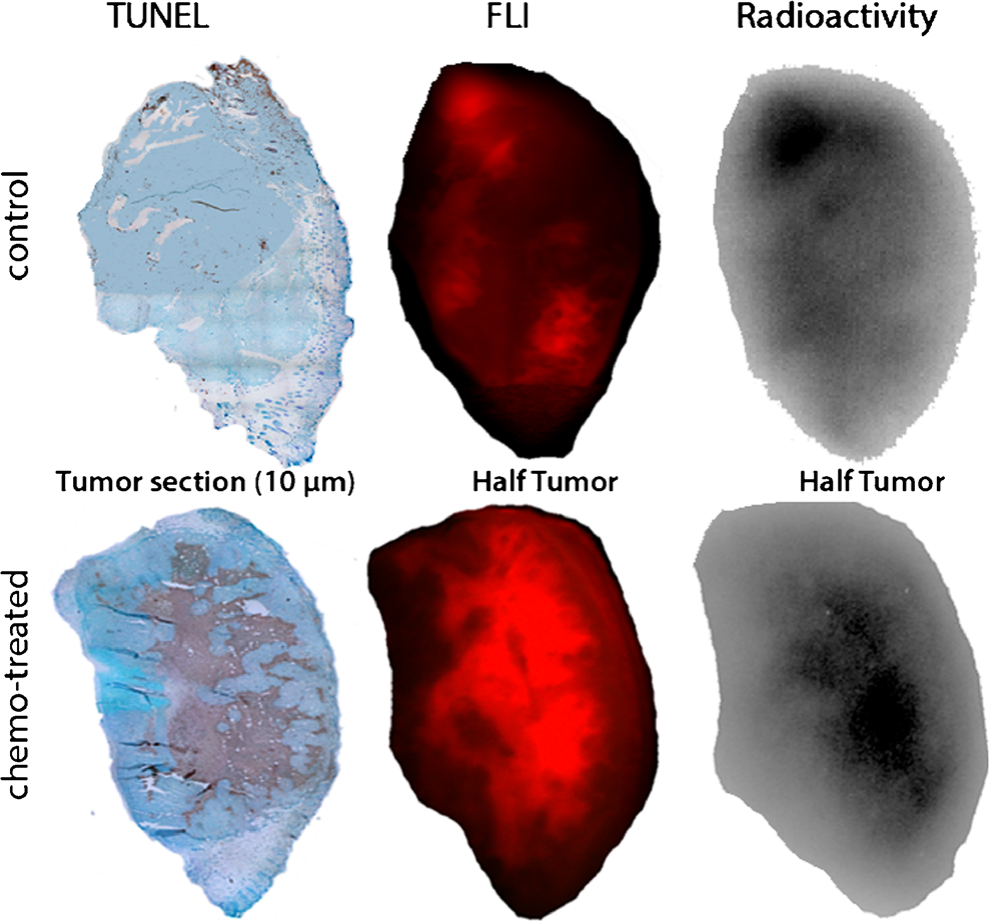
Histological and *ex vivo* analysis of [^111^In]DTPA-HQ4 injected control and chemotherapy-treated MCF-7 tumors. Tumors were dissected 24 h after probe injection and were subsequently cut in half for *ex vivo* FLI and RA analysis. The opposite half of the tumor was used for paraffin embedding and TUNEL staining (*brown* indicates the area of necrosis). *FLI* = fluorescence imaging.

Finally, tumors of chemotherapy-treated and control mice, which were injected with [^111^In]DTPA-HQ4 (10 nmol, 30–35 MBq), were dissected and cut in half to perform *ex vivo* fluorescence and radioactivity analysis. In addition, the opposite half of the tumor was processed for paraffin embedding and histological analyses. As shown in Fig. 5, the TUNEL-stained section of the untreated control tumor showed very little necrotic tissue, whereas the chemotherapy-treated tumor contained a large area of necrotic tissue (brown staining). Notably, the TUNEL staining showed co-localization with both fluorescence and radioactivity signals in tumors, confirming the specific increased necrosis retention property of [^111^In]DTPA-HQ4. For comparison, all the settings used for the different images are similar.

## Discussion

Over the last two decades, several attempts have been made to exploit disease and/or therapy-induced tumor necrosis as a diagnostic and/or prognostic biomarker of disease and to utilize necrotic tissue as a target for drug delivery. The existing necrosis imaging agents can be divided into two different groups: (non-) specific CT and MR contrast agents to enhance the natural signal and the other group consists out of mostly radiolabeled necrosis avid contrast agents (NACAs) that specifically target necrotic tissue. The latter can also be employed for therapeutic purposes, e.g., when radiolabeled with Iodine-131 [2–10, 13, 15, 23, 45–49]. It is expected that the non-specific necrosis contrast agents will fall into abeyance when compounds with high necrosis specificity become clinically available. However, thus far, only the Iodine-131 conjugated Tumor Necrosis Targeting monoclonal antibody (TNT-3), licensed by Peregrine Pharmaceuticals reached the clinical trials for therapeutic use [13, 21]. This antibody, under the brand name Cotara^TM^, has been examined in clinical trials for treatment of Glioblastoma Multiforma. However, although encouraging results have been published on the website of the manufacturer in December 2012, no follow-up studies have been reported since [50]. In addition, the necrosis avid photosensitizer hypericin, under the brand name Oncocidia, is also under investigation to enter clinical trials [14, 51, 52]. Although proof of concept has been shown for both approaches, both compounds, as mentioned earlier, suffer from major drawbacks which hampers their clinical translation.

To overcome the limitations associated with the size and possible immunogenicity of antibodies, the phototoxicity, problems with solubility and ability to aggregate, a simple, injectable and non-toxic agent that displays specific necrosis avidity is required. Recently, we demonstrated that two non-toxic constituents of the group of NIRF cyanine dyes, IRDye800CW and HQ5 carboxylate, the former being commonly used as an optical probe for biomolecular labeling [53, 54], display strong and specific necrosis avid properties *in vitro* as well as in tumor bearing mice [23]. In this study, the increased retention of NIRF cyanine dyes in tumor necrosis was examined using whole body fluorescence imaging. Although whole body optical imaging is suitable for use in small animals, it is often insufficient for assessments of deep tissues in the human body, due to the limited tissue penetration depth of NIRF light. In order to seek for potential clinical applications for our newly discovered necrosis avid cyanines, we examined the possibility to radiolabel it for *in vivo* nuclear imaging by conjugating to a chelator [55, 56]. To render HQ4 applicable for SPECT, we first conjugated HQ4 to the chelate DTPA with a linker molecule, and subsequently radiolabeled with ^111^In-Cl_3_. Following the synthesis and purification procedures, purity of 98 % was achieved. Thus, we created a molecule that targets necrosis and can be imaged using both NIRF and SPECT.

We demonstrated *in vitro* that [^111^In]DTPA-HQ4, like unconjugated HQ4, appears to specifically bind to dead cells after washing. Our findings, in the dry ice assay, show that the specificity of [^111^In]DTPA-HQ4 for necrotic cells was solely due to the presence of the HQ4 molecule, as the radiolabeled chelate [^111^In]DTPA alone and free ^111^In-Cl_3_ did not show to bind to the dead cells. Although DTPA conjugation is likely to change the chemical and physico-chemical properties of HQ4, for example by increasing its overall hydrophylicity [57], it retained its necrosis avid- and fluorescent properties. We observed a clear difference in the signal-to-background (S/B) ratio of [^111^In]DTPA-HQ4 between fluorescence- and radioactivity measurements. The reason for this discrepancy is unclear, but may involve environmental conditions that specifically affect the fluorescent properties of the molecule. Previously, Jiskoot et. al [58] reviewed the fluorescence properties of extrinsic dyes, which are strongly influenced by their environment (hydrophilic/hydrophobic or differences in pH) and/or by interactions with proteins. Such factors may also influence fluorescence signal intensity of HQ4 in an environment of living cells compared to in an environment of dead cells. However, such environmental-dependent signal intensity influences will not occur when a radiolabel, offering a quantitative readout, is utilized. Nevertheless, the observed S/B ratio of 2.5, obtained with radioactivity measurements, is theoretically sufficient for clinical translation [59]. Additional studies that determine the relationship between time, probe concentration, and S/B ratio could help to optimize the sensitivity of the measurements.

Furthermore, none of the cyanine dyes, including HQ4-DTPA, showed any acute *in vitro* toxicity in cultures of 4T1 cells. Even at the highest concentration of 20 μM (during 24 h), which is 200-fold higher compared to the concentration used to image dead cells *in vitro*, cell viability remained unaffected.

Collectively, our *in vitro* study revealed that DTPA conjugation is not deleterious to the necrosis avid properties of HQ4 and the lack of toxicity encouraging further studies with the aim of its clinical translation. Further investigations are warranted concerning the mechanism of interaction of the DTPA conjugated cyanine to specific proteins in necrotic cells. In the paper of Xie et al.[23], we showed that HQ5, a close cyanine analogue of HQ4, specifically accumulated in necrotic cells and not in apoptotic cells. Moreover, we have shown that HQ5 does not co-localize with F4/80 macrophage staining in an around the tumor, indicative of inflammation. Nevertheless, we did not yet examine these specific mechanistic properties for HQ4.

Using *in vivo* whole body optical imaging, SPECT, and *ex vivo* analysis, we confirmed the observed *in vitro* necrosis avid properties of [^111^In]DTPA-HQ4 in two tumor models: 4T1 mouse breast cancer tumor model of spontaneous necrosis and MCF-7 human breast cancer tumor model of chemotherapy-induced tumor necrosis. MCF-7 tumor model was selected to assess chemotherapy-induced necrosis due to its slow growth kinetics. MCF-7 tumor cells have a population doubling time (PDT) of approximately 38 h *in vitro* [60], thus representing a slow-growing tumor. This is in contrast to 4T1-cells, PDT +/−12 h [61], which develop necrotic cores spontaneously, and to EL4-cells, PDT +/−17 h [62], used in the previous paper [23]. Since MCF-7 cells hardly develop spontaneous necrosis during their growth, necrosis induced by chemotherapy is more evident and can be determined with higher accuracy. Moreover, MCF-7 cells are of human origin, which may also more accurately mimic the clinical situation [63].

In both mouse tumor models, the acquired optical imaging and SPECT results over time provided information on the pharmacokinetic profile of [^111^In]DTPA-HQ4. In the model of spontaneous tumor necrosis, necrotic areas in the tumor were clearly delineated using optical imaging and SPECT. No clear specific tissue interactions could be detected 6 h after injection, probably due to high quantities of unbound circulating probe in the blood at this early time point. The amount of radioactivity that retained in the 4T1 tumors remained approximately equal over time, while, it declined in most organs and body fluids, resulting in a relative increase in tumor signal intensity inside the tumor at later time points. In the chemotherapy-induced tumor necrosis model, the specific targeting of [^111^In]DTPA-HQ4 to necrotic areas of MCF-7 tumors was confirmed histologically using TUNEL staining. In this model we also observed that the kidneys of the chemotherapy-treated mice retained more radioactivity than in untreated mice. The reason for this is unknown, but it might be speculated that some necrosis may develop due to cyclophosphamide-induced renal oxidative stress which leads to perioxidative damage to the kidneys [64].

Finally, we compared the biodistribution and clearance rate of [^111^In]DTPA-HQ4 with that of the free labeled chelate ([^111^In]DTPA). We observed that both the biodistribution and the excretion rate of the two compounds were vastly different. Twenty-four hours after probe injection, [^111^In]DTPA was mainly retained in the kidneys and could hardly be detected in other organs or in the tumors, confirming our *in vitro* findings of a lack of tumor necrosis specificity. The observed accumulation of [^111^In]DTPA in the kidneys confirms findings of Boswell and colleagues [65] who also reported predominant clearance of this hydrophilic compound via this excretion route. Moreover, not only the organ distribution of these two compounds was dissimilar, the clearance rate of [^111^In]DTPA was about 8-fold faster than that of [^111^In]DTPA-HQ4. Combined, these findings indicate that both the pharmacokinetics and the tissue targeting properties of [^111^In]DTPA-HQ4 and [^111^In]DTPA are largely different and strengthen the notion that the cyanine HQ4 governs the overall necrosis avid properties of the [^111^In]DTPA-HQ4 molecule.

## Conclusion

In summary, we successfully yielded a new necrosis avid SPECT radiotracer by conjugating the necrosis avid cyanine HQ4 to DTPA, followed by radiolabeling with ^111^In-Cl_3_. We showed that, after DTPA conjugation, this newly synthesized radiotracer retained its specific necrosis targeting properties *in vitro* and *in vivo* in mouse models of spontaneous and therapy-induced tumor necrosis. The advantages of the small molecule [^111^In]DTPA-HQ4 include the following: high water solubility, NIRF property that enables deep penetration into tissues, lack of phototoxicity, and low production costs. Therefore, the necrosis avid radiotracer [^111^In]DTPA-HQ4 has the potential to be clinically translated for diagnostic-prognostic purposes and to predict early treatment outcome of anti-cancer treatments.

## Electronic Supplementary Material

Below is the link to the electronic supplementary material.

Online Resource 1 (PDF 1115 kb)
