# Supplementary material for: Pre-clinical Evaluation of a Cyanine-Based SPECT Probe for Multimodal Tumor Necrosis Imaging
Source: Mol Imaging Biol. 2016 Jun 8;18(6):905–15. doi: 10.1007/s11307-016-0972-7 (PMC5093207; doi:10.1007/s11307-016-0972-7)
Supplement: Supplementary file 1 — (PDF 1115 kb) [file 11307_2016_972_MOESM1_ESM.pdf]

Electronic Supplementary Material

Pre-clinical Evaluation of a Cyanine Based SPECT Probe for Multimodal Tumor Becrosis Imaging

Journal: Molecular Imaging and Biology

Marieke A. Stammes[1,2], Vicky T. Knol-Blankevoort[1,2], Luis J. Cruz[1], Hans R.I.J. Feitsma[3], Laura Mezzanotte[1,4], Robert A. Cordfunke[5], Riccardo Sinisi[6], Elena A. Dubikovskaya[6], Azusa Maeda[7], Ralph S DaCosta[7], Katja Bierau[8], Alan Chan[2], Eric L. Kaijzel[1], Thomas J.A. Snoeks[1], Ermond R. van Beek[1], Clemens W.G.M. Löwik [1,4].

1) *Department of Radiology, Leiden University Medical Center, Leiden, The Netherlands*
2) *Percuros BV, Leiden, The Netherlands*
3) *Department of Nuclear Medicine, Leiden University Medical Center, Leiden, The Netherlands*
4) *Department of Radiology, Erasmus Medical Center, Rotterdam, The Netherlands*
5) *Department of Clinical Pharmacy and Toxicology, Leiden University Medical Center, Leiden, The Netherlands*
6) *Institute of chemical sciences and engineering (ISIC), École polytechnique fédérale de Lausanne (EPFL), Lausanne, Switzerland*
7) *Division of Biophysics and Bioimaging, Princess Margaret Cancer Center, University Health Network, Toronto, Ontario, Canada*
8) *Department of Surgery, Leiden University Medical Center, Leiden, The Netherlands*

Corresponding author: Clemens W.G.M. Löwik, Current address: Erasmus Medical Center, Dept. Radiology, Building-room: Na-2503, Wytemaweg 80, 3015 CN Rotterdam, The Netherlands. +31 (0)10 7030797. c.lowik@erasmusmc.nl

Supplementary Figure 1: Structural characteristics and *in vitro* necrosis avid properties of HQ4 vs HQ5.

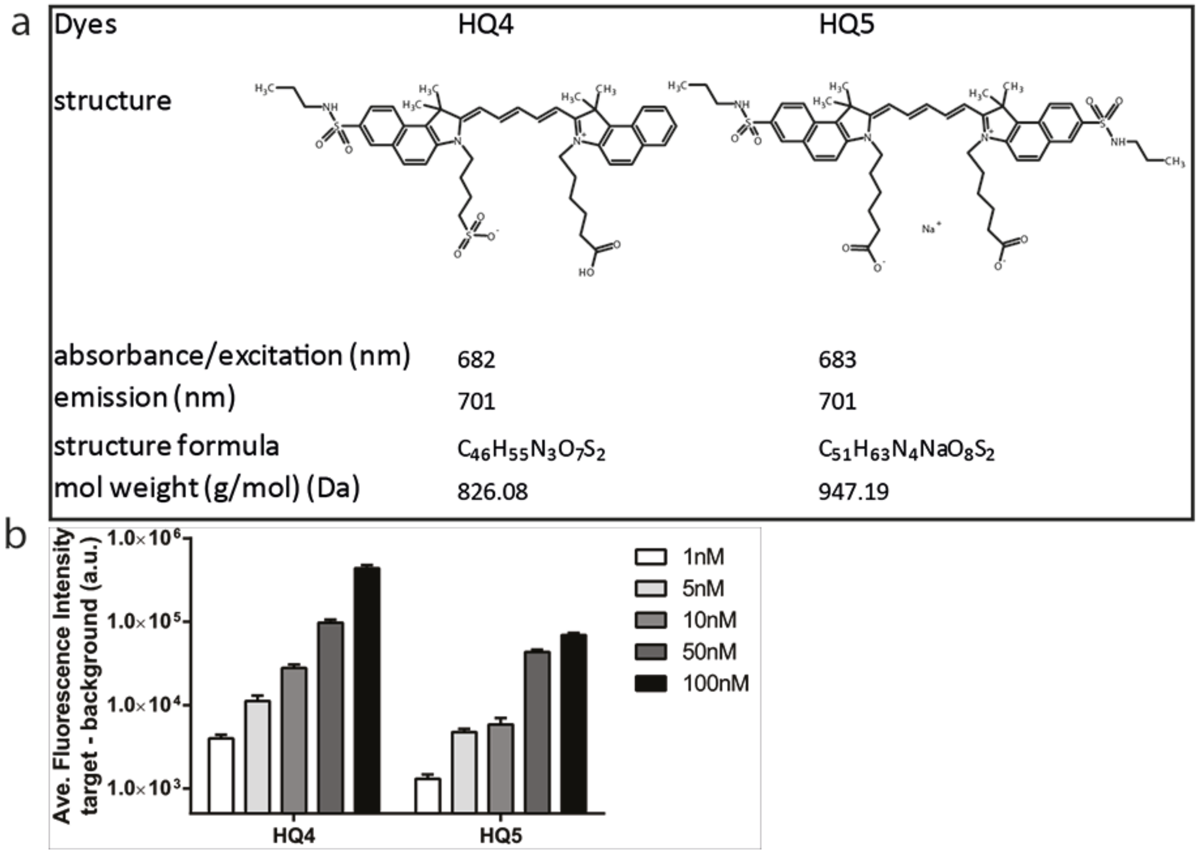

a) Chemical and structural characteristics of the carboxylated cyanine dyes HQ4 and HQ5.

b) *In vitro* necrosis targeting properties of HQ4 and HQ5 utilizing the dry ice assay. Fluorescent signal intensity was obtained from the area of dead cells in the centre of a culture well after incubation with different concentrations of HQ4 or HQ5 (1-100 nM) and is subtracted by the background signal from the area of the living cells.

Supplementary Figure 2: Reversed-phase mass spectrometry of HQ4-DTPA.

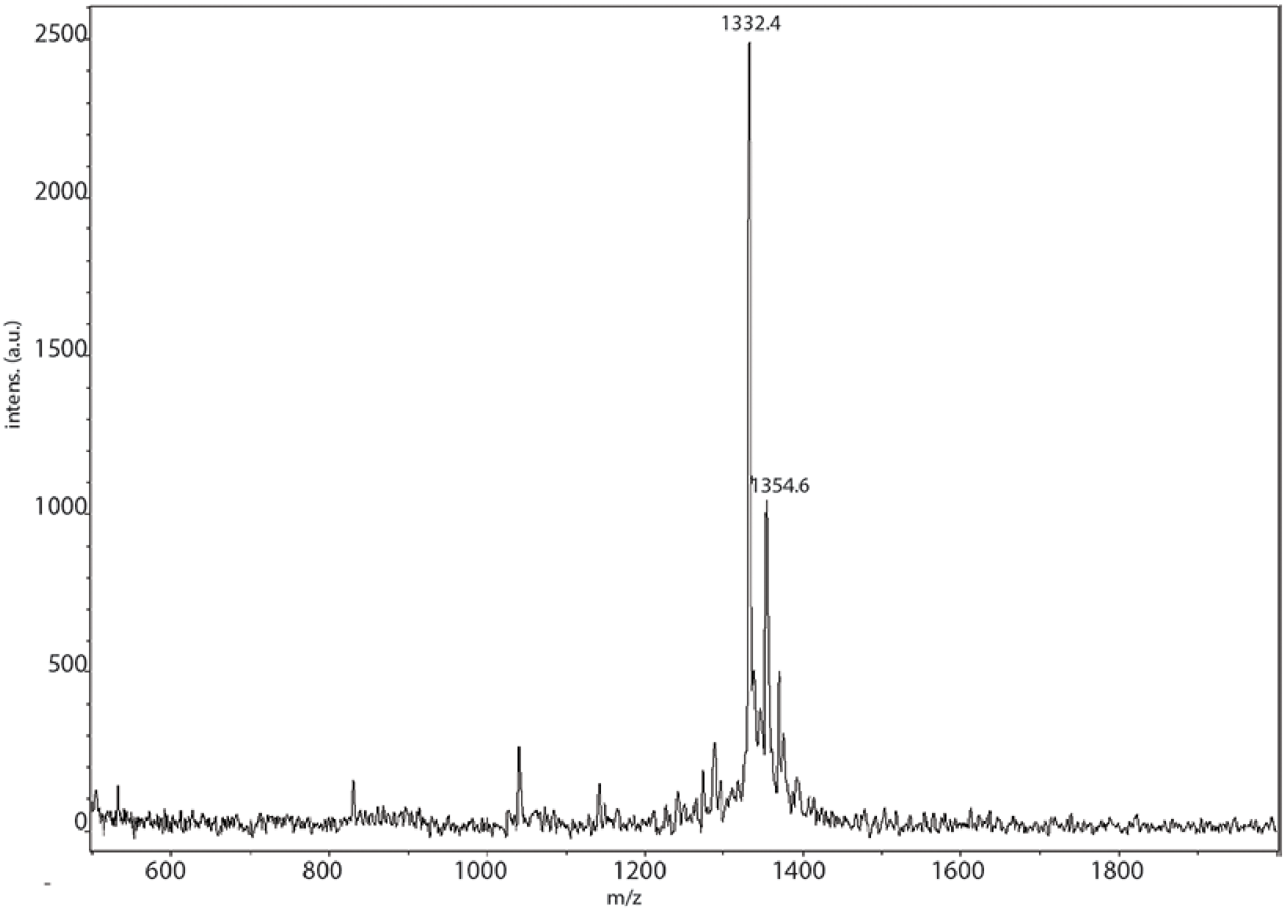

Reversed-phase chromatography showed a clear peak indicating the high grade of purity (98%) of this conjugate. Mass spectrometric analyses of HQ4-DTPA further showed the expected molecular weight (calc.: 1331.59 for $C_{66}H_{90}N_8O_{17}S_2$ and MALDI-TOF found 1332.4 [M+1]+ 1354.6 [M+Na]), indicating the high grade of purity of this conjugate.

Supplementary Figure 3: MTS cell viability assay of various agents.

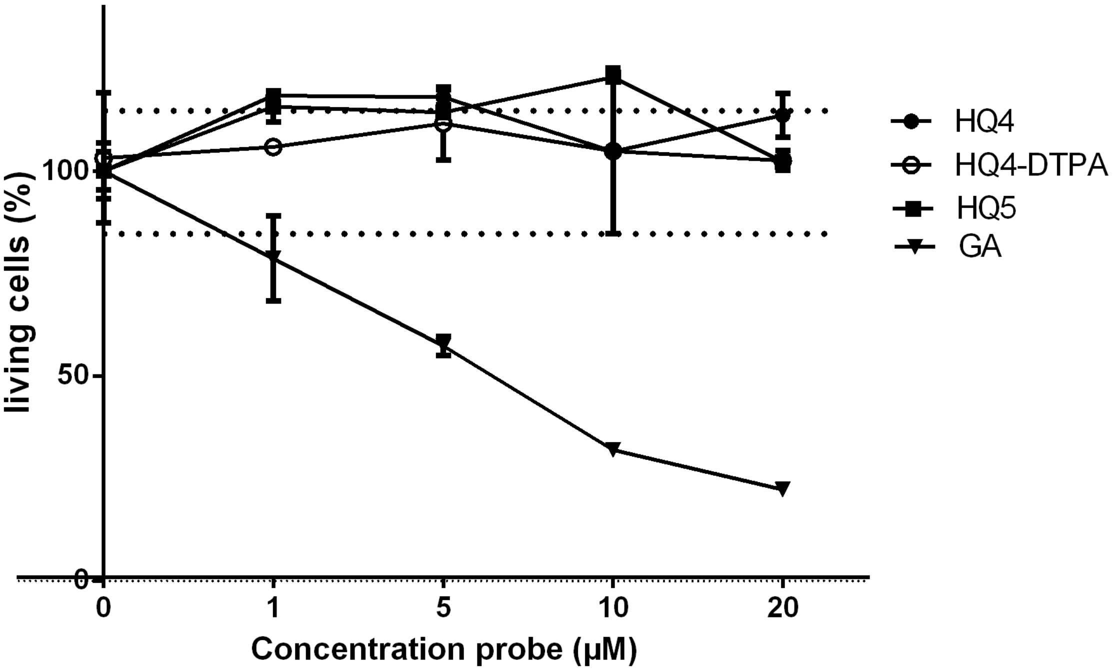

Confluent cultures of 4T1 cells were incubated for 24hr with various concentrations HQ4, HQ4-DTPA, HQ5 or the natural anti-cancer compound Gambogic Acid (GA). Relative cell viability (%) was expressed as a percentage relative to the untreated control. HQ4, HQ4-DTPA and HQ5 did not affect cell viability, whereas, GA induced cell death with an IC50 of around 6µM.

Supplementary Figure 4: Biodistribution of [^{111}In]DTPA.

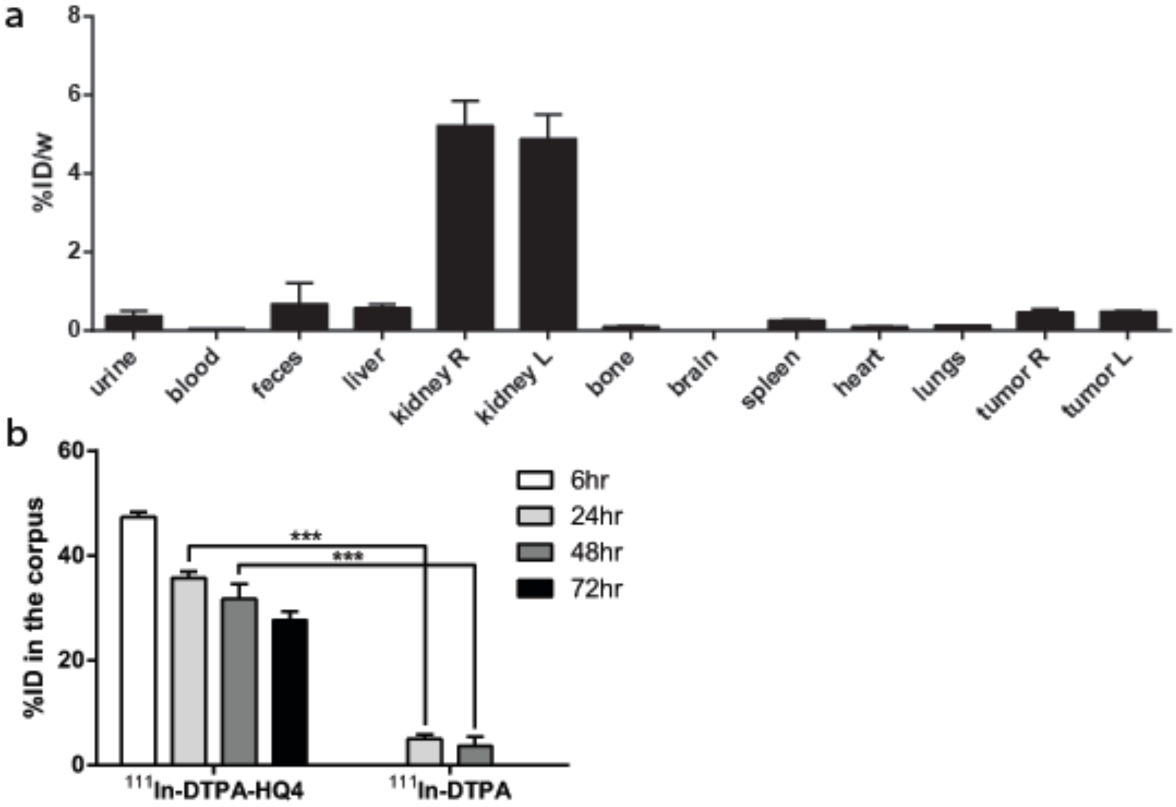

a) Biodistribution of the free chelate [^{111}In]DTPA in 4T1 tumor bearing mice. 24h after probe injection (10 µg, 30-35 MBq), mice (n=4) were sacrificed and the organs, body fluids and tumors were dissected, weighted and measured for radioactivity in a gamma counter. At each time point, the amount of radioactivity in each organ and tumor is expressed as percentage of the injected dose divided by the weight (%ID/w).

b) Total amount of remaining [^{111}In]DTPA-HQ4 and [^{111}In]DTPA in the whole mouse body (% of ID) at the indicated time points 6 to 72h after probe injection.
